# Modern Cystoscopy

**Published:** 1908-02

**Authors:** 


**A Monthly Review of Medicine and Surgery.**

*EDITOR:*

WILLIAM WARREN POTTER, M. D.

All communications, whether of a literary or business nature, books for review and exchanges, should be addressed to the editor 238 DELAWARE AVE., BUFFALO, N. Y.

VOL. LXIII. FEBRUARY, 1908. NO. 7

### Modern Cystoscopy.

DR. Bransford Lewis, professor of genitourinary diseases in the Marion Sims Medical School of St. Louis, was the guest of the Buffalo Academy of Medicine on Tuesday, January 7, 1908, at which time he read a paper on the cystoscope and demonstrated the instruments which bear his name.

The interest manifested in the subject of the paper was shown by the large attendance, more members of the academy being present than had appeared at a meeting in many months; while the paper, complete as to detail of technical manipulation of the instruments under consideration and interesting in the various phases of cystoscopy, was given the closest attention. At its conclusion Dr. Lewis was accorded an ovation which showed the sincere appreciation of his audience.

Illustrative of the advance in cystoscopy were the drawings of all the principal cystoscopes which preceded the Lewis instrument. Their technical defects were pointed out; there were also diagrammatic representations of the Lewis catheterising instrument with its various telescopic lenses by which a complete view of the bladder may be had in all directions without withdrawal of the shaft of the instrument. The drawings showed graphically the improvements which Dr. Lewis has made in his instrument; improvements which make cystoscopy less irksome to the patient, more certain for the operator and more rapid.

The paper which Dr. Lewis read was full of illustrative cases and was accompanied by photographs and skiagrams showing ureteral calculi, and one which was by far the most interesting from a cystoscopic standpoint, of three ureters. This is the case which Dr. Lewis reported at length sometime ago in which a gonorrheic resisted all forms of treatment until the operator discovered what he suspected to be a third ureteral orifice and demonstrated the third ureter by the *x*-ray, using urethral catheters through which were passed lead wires.

The demonstrations of the cystoscopes and the accessory instruments accompanying them was of the utmost interest and showed that Dr. Lewis possessed mechanical genius of high order, one of the instruments being a pair of forceps, curved and pliable, for use within the bladder, working through the shaft of the operating cystoscope and opening and closing the very small blade-tips of the instrument by a most ingenious outside contrivance.

The discussion of the paper brought out many interesting points in connection with cystoscopy, and was participated in by Dr. S. Y. Howell who detailed his experience with intravesical examination and diagnosis of obscure bladder conditions; Dr. David Wheeler, who has done much work with both the catheterising and operating instruments; Dr. J. A. Gardner, president of the section of which Dr. Lewis was the guest, and Dr. N. W. Wilson, who did work with Dr. Lewis in St. Louis four years ago, and described the appreciable improvements in technic which the originator of the instrument had made possible. Dr. J. H. Dowd discussed cystoscopy in general.

The day which Dr. Lewis spent in Buffalo was a busy one. He was breakfasted at the University Club and later was the guest of Dr. Roswell Park at the Buffalo General Hospital where he demonstrated before the senior class of the medical department of the University of Buffalo the use of the cystoscope, and catheterised the ureters in a case prior to operation. His lecture to the students was interesting and instructive, and the description of the various steps in the operation made the procedure one of picturesque dexterity. Later the visitor was entertained at luncheon, and in the evening was the guest at dinner given in his honor at the University Club by Dr. James A. Gardner, president of the section on surgery of the Academy of Medicine.

Dr. Lewis will make a tour of eastern cities prior to returning to St. Louis.

